# In cold blood: screening wild Alaskan moose (*Alces alces gigas*) for filarial nematode (Filarioidea: Onchocercidae) infections using a deep-amplicon sequencing approach

**DOI:** 10.1017/S0031182026101991

**Published:** 2026-05

**Authors:** Matthew R. Kulpa, John A. Crouse, Daniel P. Thompson, Joe L. Luksovsky, John C. Blazier, John S. Gilleard, Guilherme G. Verocai

**Affiliations:** 1 Department of Veterinary Pathobiology, College of Veterinary Medicine and Biomedical Sciences, Texas A&M University, College Station, TX, USA; 2 Alaska Department of Fish and Game, Division of Wildlife Conservation, Kenai Moose Research Center, Soldotna, AK, USA; 3 Colorado Parks and Wildlife, Grand Junction, CO, USA; 4 Texas A&M Institute of Genome Sciences and Society, Texas A&M University, College Station, TX, USA; 5 Department of Comparative Biology and Experimental Medicine, Host-Parasite Interactions Program, Faculty of Veterinary Medicine, University of Calgary, Calgary, AB, Canada

**Keywords:** *Alces alces*, blood-borne pathogens, climate change, co-infections, deep amplicon sequencing, Filarioidea, Modified Knott’s Test, *Rumenfilaria andersoni*, *Setaria yehi*

## Abstract

Moose are significant ecological, economical and cultural animals for the stakeholders of Alaska, USA. Thus, the impact of pathogens, like filarial nematodes, is a critical area of moose research. These vector-borne parasites, including *Setaria yehi*, and *Rumenfilaria andersoni*, can lead to severe health consequences (e.g., peritonitis). However, little is known about filarial nematode distribution, diversity and its associated life cycle with Alaskan moose hosts. Newly developed next-generation sequencing techniques offer the ability to efficiently screen multiple species of co-infecting filarial nematodes in a single sample and thus improve our ability to monitor and understand these parasites. Blood collected from wild moose in the Kenai Peninsula, AK, was screened using deep amplicon sequencing (DAS) with filarial nematode primers targeting the cytochrome oxidase c subunit 1 (*cox1*) gene. In addition, samples subjected to DAS were also screened using the Modified Knott’s Test (MKT). *Setaria yehi* and *R. andersoni* were detected by both diagnostic methods. Overall, 190 moose samples were tested via DAS, with filarioid DNA being detected in 51.58% (98/190) of these. Out of a subset of 138 samples, filarioid nematodes were found in 50.72% (*n* = 70) and 57.25% (79/138) via DAS and MKT, respectively. However, 18 (13.04%) co-infections were detected by DAS compared to 12 (8.70%) identified via MKT. A DAS molecular tool for surveillance has several advantages when paired with host blood collection metadata (i.e., years, season, region, host age) to better understand filarial nematode life cycle and ecology.

## Graphical abstract

**Figure:**
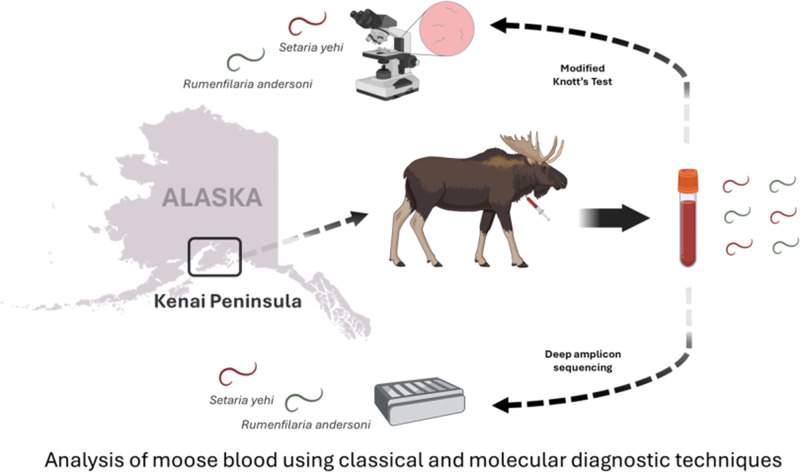

## Introduction

Since the late Pleistocene, moose, or *Alces alces* (Linneaus, 1758), have been distributed throughout the northern Nearctic. The largest extant member of the Cervidae family is primarily a solitary mammal that inhabits both the circumpolar boreal and temperate forests of North America (Peterson, 1974). There are 4 subspecies considered to reside in the continent of North America: The Alaskan moose (*A. a. gigas*) of Alaska, USA and western Yukon Territory; the northwestern moose (*A. a. andersoni*) of British Columbia to Ontario, Canada; the Shiras moose (*A. a. shirasi*) of the rocky mountains in mainly the USA; and the eastern moose (*A. a. americanus*) of eastern Ontario to the Atlantic sea board of Canada and Maine, USA (Feldhamer et al., 2003). Moose are known to have substantial impacts on their inhabited ecosystems. For example, moose can promote forest diversity through foraging, enhance soil nutrient cycling through faecal deposits and be an integral link to the terrestrial and aquatic food webs (Molvar et al., 1993; Crichton, 1998; Johnson and Rea, 2023). In addition, moose are integral to the health of local arctic communities that cohabit these geographic regions. For example, indigenous people depend on hunting moose for sustenance and their socio-cultural activities. Moose can also be drivers of economic prosperity for invested stakeholders and the general public (e.g., hunting, food and ecotourism) (Timmermann and Rodgers, 2005).

Despite wild moose importance to environmental and human stability, the epidemiological role of many pathogens is largely understudied in this host. For instance, filarial nematodes are vector-borne parasites that are known to cause devastating clinical impacts on both humans and animals but have undefined consequences for moose. There are at least four known filarial nematode genera or species known to infect moose, including *Elaeophora schneideri* Wehr and Dikmans, 1935, *Onchocerca* spp., *Rumenfilaria andersoni* Lankester and Snider, 1982 and *Setaria* species (Anderson, 2000, 2001; Feldhamer et al., 2003; Kutz et al., 2012; Benedict et al., 2023; Verocai et al., 2024; Kulpa et al., 2025b). These nematodes use distinct arthropod vectors for transmission and are found in different anatomic locations in their definitive hosts which result in various pathologies and clinical signs. Both *R. andersoni* and *Setaria* circulate throughout the blood of their hosts (Anderson, 2000; Grunenwald, 2015). While deer flies are considered putative vectors of *R. andersoni*, mosquitoes (i.e., Culicidae) are known to transmit species of *Setaria* (Anderson, 2000; Laaksonen et al., 2009a; Grunenwald, 2015). *Rumenfilaria andersoni*, a lymphatic-dwelling adult worm, has been observed to cause lymphatic obstruction leading secondary issues like lymphedema, lymphangitis and granulomatous inflammation in European ungulates (Lankester and Snider, 1982). However, no clinical signs have been reported in North American moose, but it is conceivable that this clinical manifestation has gone unnoticed at this time (Laaksonen et al., 2015; Grunenwald et al., 2016). Lastly, *Setaria* species, including *Setaria yehi* Desset 1966, mature in the abdominal cavity and can lead to peritonitis (Verocai et al., 2024). At least 9 free-ranging moose calves from Alaska have been reported to be infected with *S. yehi* that caused peritonitis and death (Kutz et al., 2012; Verocai et al., 2024). In addition, other species of *Setaria*, like the European parasite *Setaria tundra* Bain, 1974, have caused severe outbreaks of peritonitis infections in Finnish reindeer (i.e., the same mammalian species as North American caribou) and moose (Laaksonen et al., 2007, 2009b; Laaksonen, 2010). While *S. tundra* is not known to be present in North America, there is speculation the parasite could have been introduced and established through translocated semi-domesticated reindeer from Eurasia into Alaska over a century ago (Finstad et al., 2006; Verocai et al., 2013, 2024).

The knowledge gap between North American filarial nematodes and their relationship to *A. alces*, undermine our potential to conserve these culturally, ecologically and economically significant mammals. Specifically, *Setaria* species are a notable threat to Alaskan moose health. Moose populations have already been shown to be vulnerable to *S. yehi* associated disease, but moreover, these conditions are expected to only worsen. *Setaria*-caused peritonitis outbreaks are linked to warmer and longer summers, which can lead to increased arthropod vector exposure time. This means that temperature warming caused by climate change will only perpetuate the risk of *Setaria* infection to moose (Laaksonen et al., 2010). There is also no information on how climate change is affecting sympatric filarial nematode species in Alaska, like *R. andersoni* (Kutz et al., 2012; Grunenwald et al., 2016). Recently collected blood samples from the Alaskan moose of the Kenai Peninsula, AK can be used to screen filarial nematode species infecting these hosts. By using recently developed deep amplicon sequencing (DAS) tools (Huggins et al., 2024), blood samples with individual and/or co-infected filarial parasites can be detected (Kulpa et al., 2025a). This work will help lay foundational knowledge to filarial nematode life cycles, biogeographic distributions and unrealized diversity in relation to Alaskan moose hosts and for broader parasitology-related applications in the future.

## Materials and methods

### Moose blood collection and metadata

**Figure 1.**
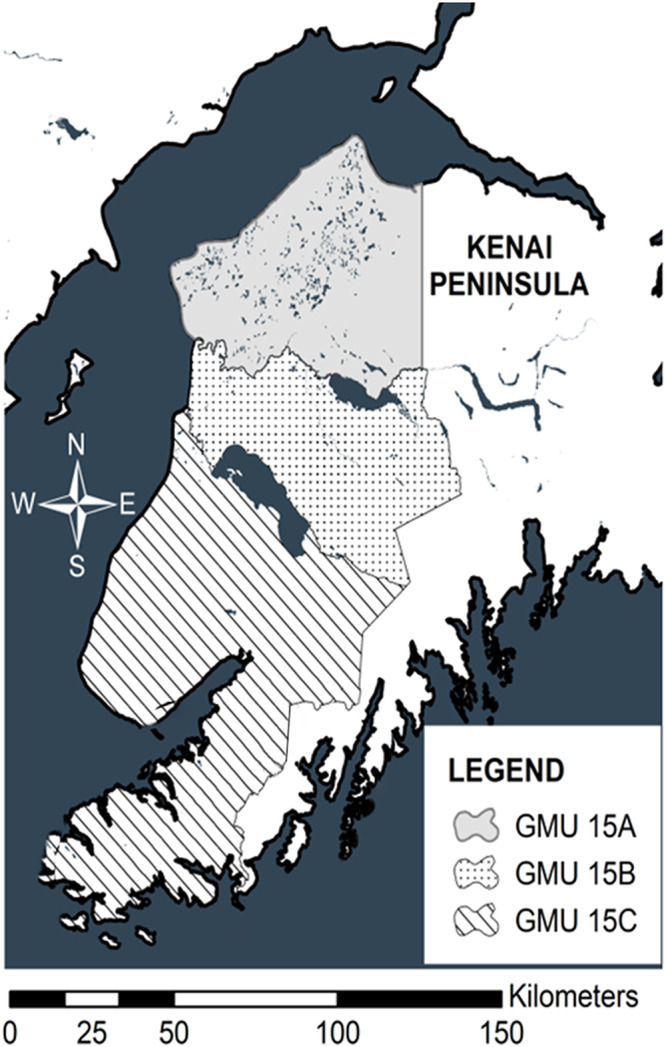
A map of Kenai Peninsula, Alaska. The legend defines each region where moose blood was collected from on the western side of the peninsula. This includes 15A the northernmost region (*n* = 80), 15B the central region (*n* = 93) and 15C the southernmost region (*n* = 17). Figure 1 long description.

All moose blood samples were collected from wild free-ranging moose of the western Kenai Peninsula, AK in Game Management Unit (GMU) 15 from 2015 to 2022. This included a total of 190 moose samples from the 3 subunits in GMU 15: 15A, 15B and 15C (Fig. 1). Detailed information on every sample, including year, season, region and age can be found in the Supplementary Materials. Adult female and 10-month calves (male and female) were chemically immobilized via aerial darting following protocols outlined in Thompson et al. (2024). Blood samples were collected into 6 mL plastic trace element whole-blood tubes (K2 EDTA; BD Vacutainer^®^; Becton, Dickinson and Company) by jugular venipuncture. Collected blood samples were stored in −80 °C freezer until further processing.

### Modified Knott’s Test

Moose blood samples from 2019 to 2022 were analysed using a Modified Knott’s Test (MKT), a diagnostic approach that involves morphological identification of microfilariae (mf). As previously described (Verocai et al., 2024), A 0.1% methylene blue dye was added to the sediment to allow for mf visualization and genus or species-level identification.

### DNA extraction and sequencing

Frozen moose blood samples were initially thawed at 21° C in a thermomixer at 300 rpm for 30 minutes. Once complete, 200 μL of each sample was aliquoted and placed into a Maxwell^®^ RSC Instrument (Promega Corporation, Madison, WI, USA) for automated DNA extraction using a Maxwell^®^ RSC Tissue DNA Kit (Promega Corporation, Madison, WI, USA), according to the manufacturer’s protocol. All samples were then stored at −80 °C until further processing.

Blood samples were prepped and sequenced according to previously outlined methods (Kulpa et al., 2025a). This included using Oxford Nanopore Technologies (ONT) targeting a ∼700 base pair (bp) fragment of the cytochrome oxidase subunit I gene of the mitochondrial genome of filarial nematodes (COINT (forward) 5’- TGATTGGTGGTTTTGGTAA-3’ and COINT (reverse) 5’- ATAAGTACGAGTATCAATATC-3’) as in previous studies (Kulpa et al., 2025b). Native Barcode ligation comprised a unique barcode (Table S2) using a Native Barcoding Kit 96 V14 and carried out according to its specified protocol ONT website (SQK-NBD114.96). This final prepped library was added to the R10.4.1 flow cell (FLO-PRO114M) and loaded into a MinION™ platform at the Molecular Genomics Core at the Texas A&M Institute of Genome Sciences and Society (TIGSS).

### Bioinformatics analyses

Raw signal data from Oxford Nanopore sequencing was base-called using Dorado v0.8.2 with the super-accurate base-calling model v5.0.0 (dna_r10.4.1_e8.2_400bps_sup@v5.0.0). Reads were demultiplexed using Dorado Demux with the barcode-both-ends option to retain reads with double-ended barcodes. Filtered reads were then clustered and consensus-called using *amplicon_sorter* (https://github.com/avierstr/amplicon_sorter) using parameters -min 400 -max 800 -ar -np 48 -aln (Vierstraete and Braeckman, 2022) and 20 000 reads were processed per sample. To ensure samples with low levels of mf were not missed, no minimum read threshold was established. However, no consensus read clusters fell below 22 reads in our study and all consensus sequences were visually inspected to validate their identity and quality. All analyses were performed on the Grace computing cluster at Texas A&M University. The following command was used to download applicable sequences from NCBI’s GenBank ‘esearch -db nucleotide -query “cox1[Gene] AND (Brugia OR Wuchereria OR Onchocerca OR Dirofilaria OR Loa OR Mansonella OR Acanthocheilonema OR Setaria OR Dipetalonema OR Litomosoides OR Parafilaria OR Foleyella OR Cercopithifilaria OR Chandlerella OR Elaeophora OR Stephanofilaria OR Monanema OR Pelecitus OR Rumenfilaria) AND mitochondrion[filter]”\| efetch -format fasta > all_filarial_cox1.fasta.’ Blast analysis can be found in Supplementary Materials.

### Statistical analyses

Comparisons between MKT and DAS were done using a paired McNemar’s test. These comparisons were made by various categories including year (2019–2022), season (spring and fall), region (A, B and C) and host age (calf and adult). Additionally, a simple linear regression was done to compare the proportion of DAS reads and the proportion of estimated mf/mL for each species.

## Results

### Overview of deep amplicon sequencing analysis and reads

Consensus sequences were 649 bp in length after being appropriately trimmed and all samples, if positive for filarial DNA, comprised *R. andersoni* and/or *S. yehi*. All consensus *Rumenfilaria* sequences were identical to one another. On the other hand, consensus *Setaria* sequences had a unique single-nucleotide polymorphism (SNP) at the 613 bp region. Regardless of the *Setaria* base-pair variant (i.e., C, T or gap), each of the 3 haplotypes overlap in time, season, geographic region and age (Table 1).

**Table 1.** Comparing *S. yehi* sequences that differentiate at the 613 base pair position across individual moose positive for *S. yehi* sequence variations (SVs) by year, season, age, region and age Table 1 long description.

YEARS	SV 1 (C)	SV 2 (T)	SV 3 (GAP)
2019	3	2	0
2020	26	12	1
2021	0	0	0
2022	5	3	0
**SEASON**			
Spring	31	15	1
Fall	3	2	0
**REGION**			
15A	18	5	1
15B	15	12	0
15C	1	0	0
**AGE**			
Calf	24	9	1
Adult	10	8	0
**TOTAL**	**34**	**17**	**1**

*Setaria yehi* sequence 1 comprises a C nucleotide, *S. yehi* sequence 2 comprises a T nucleotide, and *S. yehi* sequence 3 has a gap in this position.

**Figure 2.**
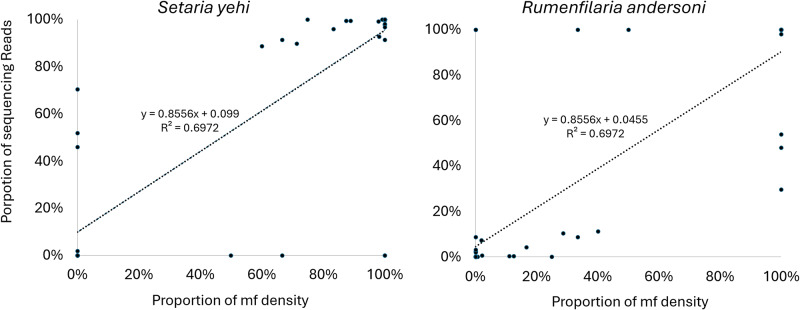
Scatter plot with a linear regression. The proportion DAS reads versus the proportion of mf density of each filarial species (i.e., *Rumenfilaria andersoni; Setaria yehi*) from filarial positive samples (*n* = 67). Figure 2 long description.

Out of the 190 moose blood samples tested, filarial nematode DNA was found in 98 (51.58%) samples. Of these, DAS detected *R. andersoni* DNA in 64 samples (33.68%) and *S. yehi* DNA in 52 (27.37%), including single infections and co-infections. *Rumenfilaria andersoni* produced roughly 2568 (24 – 16 678 reads) and *S. yehi* produced 5902 (22 – 16 944 reads) mean average reads when detected in a sample. Positive samples for both DAS and MKT (*n* = 67) were plotted against each other using the proportion of estimated mf density and proportion of reads for each species. A statistically significant linear relationship was found between the 2 diagnostic tests (*n* = 67; F_1,65_ = 184.40, P < 0.001, R^2^ = 0.74; Fig. 2). Out of these 190 blood samples, DAS found 18 samples (9.5%) were co-infections by *S. yehi* and *R. andersoni*. Co-infections produced a mean average of 297 (24 – 3692) *R. andersoni* reads and 5946 (22 – 16 459 reads) *S. yehi* reads.

### Comparing Modified Knott’s Test and deep amplicon sequencing filarial detection

**Figure 3.**
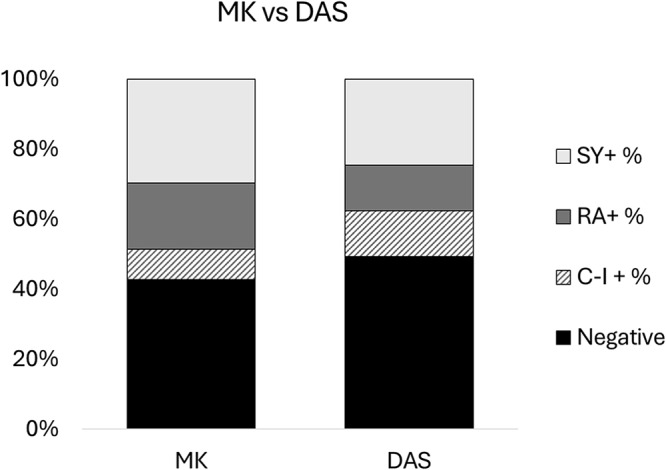
Comparing the difference in filarial species detection between MKT) and DAS diagnostic approaches. Filarial species detection is broken down into *S. yehi* only positive (SY +), *R. andersoni* only positive (RA +), co-infected (CI +) and negative samples. The mean average percentage of filarial detection is shown between MKT (left) and DAS (right). Figure 3 long description.

Two species of filarial nematodes were identified through MKT and DAS, *R. andersoni* and *S. yehi*. A total of 138 samples were both subjected to MKT and DAS diagnostic approaches. MKT detected more filarial infections than DAS (57.25% and 50.72%, respectively) and statistical analysis revealed a borderline significant difference between MKT and DAS approaches (P = 0.05). However, DAS detected 6 additional co-infected samples (i.e., both *R. andersoni* and *S. yehi*) compared to MKT (Table 2, Fig. 3).

**Table 2.** Table comparing MKT and DAS diagnostic approaches and their ability to detect filarial infection in moose blood by year, season, age, region and age Table 2 long description.

	Modified Knott’s test				Deep amplicon sequencing			
YEARS	Negative	*Setaria yehi*	*Rumenfilaria andersoni*	Co-infections	Negative	*Setaria yehi*	*Rumenfilaria andersoni*	Co-infections
2019 (*n* = 12)	4 (16.00%)	7 (58.33%)	3 (25.00%)	2 (16.67%)	5 (41.67%)	5 (41.67%)	3 (25.00%)	1 (8.33%)
2020 (*n* = 84)	30 (35.71%)	38 (45.24%)	24 (28.57%)	8 (9.52%)	36 (42.86%)	39 (46.43%)	24 (28.57%)	15 (17.86%)
2021 (*n* = 10)	3 (30.00%)	0 (0.00%)	7 (70.00%)	0 (0.00%)	5 (50.00%)	0 (0.00%)	5 (50.00%)	0 (0.00%)
2022 (*n* = 32)	22 (68.75%)	8 (25.00%)	4 (12.50%)	2 (6.25%)	22 (68.75%)	8 (25.00%)	4 (12.50%)	2 (6.25%)
**SEASON**								
Spring (*n* = 116)	52 (44.83%)	46 (39.66%)	28 (24.14%)	10 (8.62%)	58 (50.00%)	47 (40.52%)	28 (24.14%)	17 (14.66%)
Fall (*n* = 22)	10 (45.45%)	7 (31.82%)	10 (45.45%)	2 (9.09%)	10 (45.45%)	5 (22.73%)	8 (36.36%)	1 (4.55%)
**REGION**								
15A (*n* = 55)	20 (36.36%)	24 (43.64%)	20 (36.36%)	9 (16.36%)	24 (43.64%)	24 (43.64%)	17 (30.91%)	10 (18.18%)
15B (*n* = 67)	28 (41.79%)	28 (41.79%)	14 (20.90%)	3 (4.48%)	32 (47.76%)	27 (40.30%)	16 23.88%)	8 (11.94%)
15C (*n* = 16)	11 (68.75%)	1 (6.25%)	4 (25.00%)	0 (0.00%)	12 (75.00%)	1 (6.25%)	3 (18.75%)	0 (0.00%)
**AGE**								
Calf (*n* = 63)	24 (38.10%)	35 (55.56%)	11 (17.46%)	7 (11.11%)	27 (42.86%)	34 (53.97%)	12 (19.05%)	10 (15.87%)
Adult (*n* = 75)	35 (46.67%)	18 (24.00%)	27 (36.00%)	5 (6.67%)	41 (54.67%)	18 (24.00%)	24 (32.00%)	8 (10.67%)
**TOTAL**	**59 (42.45%)**	**53 (38.41%)**	**38 (27.54%)**	**12 (8.70%)**	**68 (49.28%)**	**52 (37.68%)**	**36 (26.09%)**	**18 (13.04%)**

Filarial DNA detection for each diagnostic approach is broken down by *S. yehi*-positive samples, *R. andersoni*-positive samples, and co-infections.

### Comparing Modified Knott’s Test and deep amplicon sequencing by year

**Figure 4.**
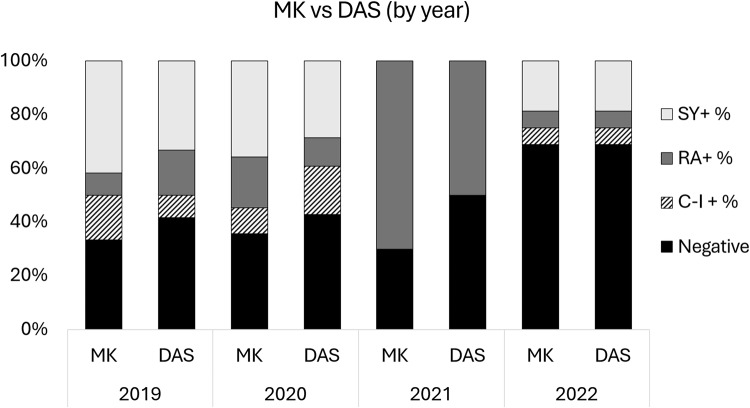
Bar graphs comparing the difference in filarial species detection between MKT and DAS by year (i.e., 2019–2022). Filarial species detection is broken down into *S. yehi* only positive (SY +), *R. andersoni* only positive (RA +), co-infected (CI +) and negative samples. Figure 4 long description.

A total of 138 samples were processed with 12 in 2019, 84 in 2020, 10 in 2021 and 32 in 2022. MKT revealed 8, 54, 7 and 15 positive filarial samples and DAS revealed 7, 48, 5 and 10 positive filarial samples for 2019, 2020, 2021 and 2022, respectively. All positive samples from 2021, using MKT or DAS, detected *R. andersoni*. All other years had a mix of *S. yehi* and/or *R. andersoni* positives. Direct comparisons of MKT and DAS were not significant for 2020 (P = 0.06) and 2021 (P = 0.25) samples. Data from 2019 and 2022 was in complete agreement between diagnostic tests (Fig. 4).

### Comparing Modified Knott’s Test and deep amplicon sequencing by season

**Figure 5.**
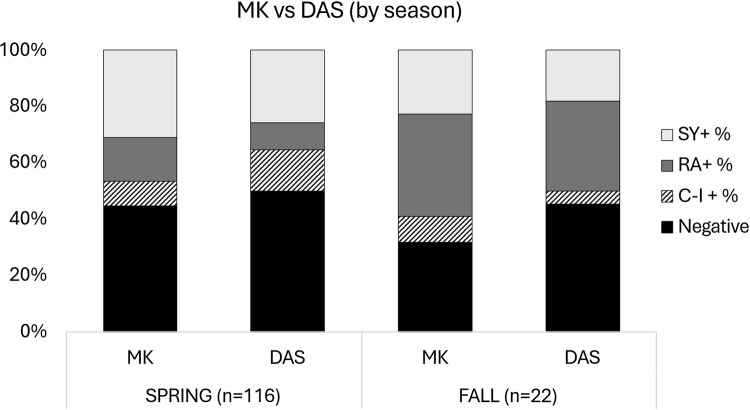
Bar graphs comparing the difference in filarial species detection between MKT and DAS by season (i.e., spring and fall). Filarial species detection is broken down into *S. yehi* only positive (SY +), *R. andersoni* only positive (RA +), co-infected (CI +) and negative samples. Figure 5 long description.

Most blood samples used for diagnostic test comparisons (2019–2022) were collected in the spring (*n* = 116) compared to the fall (*n* = 22). MKT found 64 and 15 positive filarial samples and DAS found 58 and 12 positive filarial samples for spring and fall, respectively. Direct statistical comparisons of MKT and DAS by season showed there was no statistical significance in spring (P = 0.08) or fall season (P = 0.25) (Table 2, Fig. 5).

### Comparing Modified Knott’s Test and deep amplicon sequencing by region

**Figure 6.**
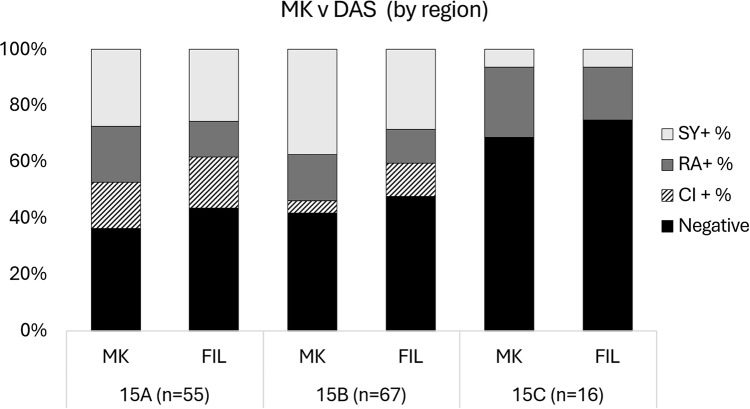
Bar graphs comparing the difference in filarial species detection between MKT and DAS by region (i.e., 15A, 15B and 15C). Filarial species detection is broken down into *S. yehi* only positive (SY +), *R. andersoni* only positive (RA +), co-infected (CI +) and negative samples. Figure 6 long description.

Out of the 138 samples that were processed, 55 samples were from GMU 15A, 67 samples were from 15B and 16 samples were from region 15C. MKT revealed 35, 39 and 5 positive filarial samples and DAS revealed 31, 35 and 4 positive filarial samples for regions 15A, 15B and 15C, respectively. When comparing MKT and DAS, no significant differences were found among regions 15A (P = 0.11), 15B (P = 0.15) or 15C (P = 1) (Table 1, Fig. 6).

### Comparing Modified Knott’s Test and deep amplicon sequencing by host age

**Figure 7.**
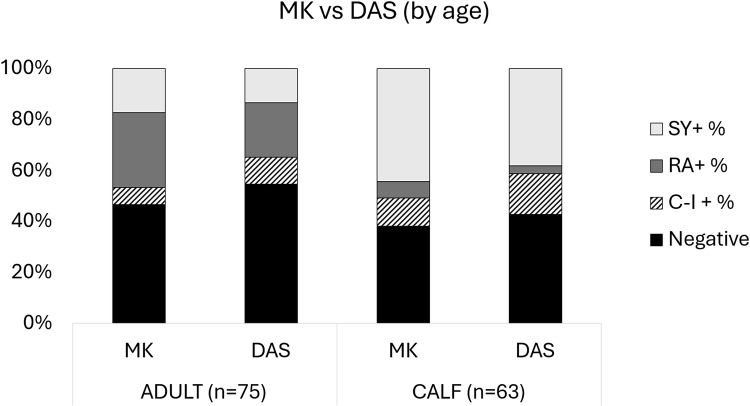
Bar graphs comparing the difference in filarial species detection between MKT and DAS by age (i.e., calf and adult). Filarial species detection is broken down into *S. yehi* only positive (SY +), *R. andersoni* only positive (RA +), co-infected (CI +) and negative samples. Figure 7 long description.

Out of 138 samples that were processed, 63 samples were collected from calves and 75 samples were collected from adults. MKT revealed 39 and 40 positive filarial samples and DAS revealed 36 and 34 positive filarial samples for calves and adults, respectively. When comparing MKT and DAS, no significant differences were found by calves (P = 1) and adults (P = 0.09). However, adults had substantially higher numbers of *R. andersoni*-positive samples compared to *S. yehi*-positive, and calves had substantially higher numbers of *S. yehi*-positive samples compared to *R. andersoni*-positive, regardless of diagnostic test (Table 2, Fig. 7).

### Using deep amplicon sequencing for filarial detection in moose blood samples (2015–2018)

Moose blood samples collected from 2015 to 2018 (*n* = 52) were used for DAS with no MKT comparisons (Supplementary materials). All samples were collected from adult female moose in the month of November (fall). A total of 28 samples, or 53.84%, were positive for filarial DNA and all these sequences were identified as *R. andersoni*.

## Discussion

### Advantages of deploying a deep amplicon sequencing tool for studying filarial nematodes

Filarial nematodes pose a unique and complex One Health crisis for cervids and humans alike in northern arctic communities like Kenai Peninsula, Alaska. Recent outbreaks of peritonitis caused by *Setaria* have endangered both moose calves in Alaska and reindeer in Finland. Moreover, there are troubling reports of indigenous Canadian hunters abandoning caribou harvests due to damage caused by *Setaria* infection (Kutz, 2025). This could have a serious impact on communities that rely on traditional foods like cervids for their livelihood. Despite these harmful observations and trends, little research has been done to expand our knowledge of filarial nematode diversity, geographic distribution and host-/vector-associations. This study of filarial nematodes infecting Alaskan moose was developed to employ a novel molecular surveillance approach that would help fill in these knowledge gaps and aide in monitoring potential One Health threats to wildlife and northern arctic communities.

Currently, at least 2 filarial species are known to infect moose in the Kenai Peninsula, *R. andersoni* and *S. yehi*, and there could be additional filarial species posing a health risk to cervids. Since multiple species are problematic, a diagnostic approach that detects co-infections is critical to gather foundational knowledge of parasite life cycle, distribution and diversity. This provides DAS with some advantages compared to common molecular approaches like quantitative PCR (qPCR), which are used to target specific filarial species depending on primer design (Verocai et al., 2024). On the other hand, MKT is a classical microscopic technique that directly identifies mf, allowing for the ability to diagnosis co-infected samples. However, this is a laborious and time-consuming method that requires specialized taxonomic training. The increasing affordability, portability and functionality of DAS provide a practical, high-throughput alternative to a MKT. However, it should be noted that DAS is not a replacement to classical diagnostic approaches for detecting filarial nematodes. In fact, MKT may still be more sensitive at detecting low levels of microfilaremia, especially in single species infections.

While classical techniques did identify more filarial positive samples, DAS outperformed MKT to detect co-infections. When taking into consideration these samples with undetected filarial species, the amount of overall filarial DNA uncovered by DAS is much closer to the amount of filarial positive samples microscopically diagnosed in this study. There is also less risk of human induced error or bias in identifying mf, which could explain some of the discrepancy. Another possible reason for incongruity could be due to blood collection time, location and random chance. Blood samples can have very low presence of worms or DNA and visual inspection may be the only way to determine infection. Moreover, the physical subset sample used for either MKT or DAS analysis from a larger blood collected tube may not comprise the same amount of mf or DNA. It should also be reiterated that the MKT requires 1 mL of blood and deep amplicon sequencing requires 200 µL.

Another advantage of using DAS for filarial DNA detection is its ability to use frozen archival samples (−80 °C) from as far back as 2015. Freeze–thaw methods can cause physical damage and alter the appearance of mf (Sturiale et al., 2025). This means classical techniques like MKT, which are dependent on morphology, could be susceptible to misdiagnosis when using frozen samples. Current evidence shows little effect on diagnostic accuracy between frozen and fresh storage of *S. yehi* and *R. andersoni*, when frozen samples undergo a single freeze–thaw cycle (Verocai et al., 2024). However, it is unknown if multiple freeze–thaw cycles or increased amount of time frozen could alter these results. Regardless, evidence indicates that decade stored frozen blood does not limit detection of filarial nematode by DAS, and these types of samples will be useful to investigate the temporal dynamics of filarial host infections. Although not explored in the current study, DAS has the distinct advantage to utilize skin or other animal tissues. As previously noted, *Onchocerca* and *Elaeophora* are 2 genera of filarial nematodes that have mf that dwell within the skin of cervids and other mammals. While this study is limited in recovering skin-dwelling mf, future work that wishes to study these parasites could use a flexible diagnostic technique like DAS.

Other molecular approaches like droplet digital PCR (ddPCR) have been used for surveillance of filarial nematodes among wild North American cervids, like *S. yehi* and *Onchocerca* species of caribou (Thomas et al., 2025). The use of ddPCR was substantially more sensitive for detection of these types of parasites compared to other targeted approaches like qPCR. Indeed, ddPCR would be most useful to in studies that need to target a specific filarial species with low concentration of DNA. On the other hand, our DAS approach is most useful for detecting multiple species of filarial nematodes and capturing unknown population diversity. For example, not only were co-infections detected, but molecular analysis was able to characterize intraspecies diversity in consensus *S. yehi* sequences. A single SNP at the 613 bp region was detected among *S. yehi*. At this time, we were unable to uncover any environmental, temporal, or host factors that may influence haplotype selection. While the implications for *S. yehi* intraspecies diversity require additional exploration, the finding highlights the broad genetic and diagnostic applications of such a precise molecular tool, particularly in the fields of human and animal health, evolution and conservation.

In addition, DAS has much broader implications for understanding host–parasite assemblages. In North America, both moose and white-tailed deer are definitive hosts of *R. andersoni* (Grunenwald et al., 2016). This parasite is also known to infect reindeer, which is the same species as the North American caribou (i.e., *Rangifer tarandus*). Nevertheless, there are no current reports of *R. andersoni* parasitizing caribou. North American moose, white-tailed deer, mule deer and caribou are all definitive hosts of *Setaria* species, often attributed as *S. yehi*. However, there are questions to the relative diversity of *Setaria* being transmitted among host cervids throughout North America and this is currently being investigated (Kulpa et al., 2024). DAS has the potential to fill these gaps in knowledge without losing information regarding animals that are co-infected with multiple species of filarial nematodes. Moreover, DAS capabilities extend far beyond the scope of this study to explore host–parasite relationships and their larger biogeographical distribution.

### Sequenced reads and microfilariae density

Blood samples comprising filarial DNA had on average much higher read counts for *S. yehi* (5902 reads) than for *R. andersoni* (2568 reads). Co-infected blood samples also had on average more *S. yehi* (5566 reads) than *R. andersoni* (671 reads) reads. There are 2 likely reasons for this pattern in the data. Firstly, when looking at sample data that also comprised Modified Knott’s Tests, *S. yehi* also had on average considerably higher microfilaremia compared to *R. andersoni* (1052 and 155 mf/mL, respectively). Therefore, a large discrepancy in reads by filarial species is expected due to quantitative differences. The second factor that could be influencing these numbers is biases in the species of filarial nematode being sequenced. Recent work has shown a disproportionate amount of reads sequenced between *Dirofilaria immitis* and *Brugia pahangi* DNA despite equal amounts of larvae being amplified (Kulpa et al., 2025a). It is unclear why these preferences exist, but it is speculated that it could be due to primer binding efficiencies even though these primers can broadly amplify the DNA of most known Onchocercidae parasites, including *Rumenfilaria* and *Setaria* species (Lefoulon et al., 2015). However, it is important to note that only a small subset of collected blood samples contained co-infections and previous biases were largely noticed in pools comprising more than one species of filarial nematode (Kulpa et al., 2025a). While amplification issues could exist in naturally infected samples with only a single filarial species, further evidence is needed to support these conclusions. Temporal factors could also change the quantity of mf in a collected blood sample. Both seasonality and periodicity influence mf density in the peripheral blood vessels of hosts (Ansari, 1964; Nelson, 1966; Laaksonen et al., 2009a). By matching the peak activity of their associated arthropod vectors, *Setaria* and *Rumenfilaria* can increase their likelihood of being transmitted to a new susceptible host. Moreover, the anatomical location of a blood drawing (e.g., the jugular vein) will be affected by these rhythmic changes of mf. Overall, either one of these factors or a combination of them, could explain the differences in mf/mL and read counts seen in our study.

### Filarial nematode epidemiology in the face of climate change and human influence

Collecting blood for filarial surveillance was important for 2 main reasons. Firstly, foundational research on filarial nematodes infecting wild subarctic mammals, like moose, is severely lacking. Thus, our study helps fill an important knowledge gap to help preserve wildlife integral to the Alaskan ecosystem and human health and culture. Secondly, climate change is severely impacting the distribution and pathogenicity of filarial nematodes across the globe. As discussed previously, filarial nematodes causing serious disease outbreaks, like peritonitis, has been well-documented in wild ungulates in Finland and Alaska (Laaksonen et al., 2007, 2010; Laaksonen, 2010; Kutz et al., 2012; Verocai et al., 2024). These disease outbreaks are expected to be caused by warming temperatures that prolong host exposure-time and increase habitat ranges of arthropod vectors which transmit filarial nematodes (Patz et al., 2000; Hoberg et al., 2008; Laaksonen et al., 2010). In addition, environmental perturbations, like climate change, can disrupt and modify host–parasite–vector assemblages. Host switching is believed to be most common during events of biotic expansion, where previously isolated parasites and hosts come in to contact with each other (Brooks and Hoberg, 2007; Hoberg and Brooks, 2008; Kutz et al., 2014). Therefore, the North American ecosystem is expected to be susceptible to parasite emergence and re-emergence with greater risks of disease outbreaks. To begin to comprehend these systemic epidemiological shifts, improvement in monitoring filarial nematodes is needed, especially neglected wildlife parasites such as *Setaria* and *Rumenfilaria*.

While no DNA of unreported filarial species infecting Alaskan moose hosts was recovered, there is recent evidence and discussion of human-mediated translocation of animals infected with *S. tundra* (Paul, 2009; Kulpa et al., 2024; Verocai et al., 2024). *Setaria tundra* has previously caused significant peritonitis outbreaks in reindeer of the Fennoscandian Peninsula and is a concern to infect and cause similar conditions in the declining population of North American caribou (Laaksonen et al., 2007). Furthermore, there is at least one report that details a *Setaria*-associated peritonitis outbreak in European moose (Nygren, 1990; Laaksonen et al., 2009b). Future work should investigate if *S. tundra* has established itself in the North American ecosystem and, if so, its impact among wild cervids thus far. It is also worth noting that unrealized cryptic diversity of wildlife parasites is well recognized, particularly among filarial nematodes infecting wild cervids of the Nearctic (Verocai et al., 2012; Kulpa et al., 2021, 2022, 2025b; Benedict et al., 2023). Thus, disentangling filarial nematode diversity is complex and could prove problematic for future researchers to discern which parasite species are emerging, introduced or unrealized.

### Exploring life cycle patterns of Setaria and Rumenfilaria

Despite the commonality of *S. yehi* and *R. andersoni* infections and co-infections in Alaska, there is little scientific literature or established knowledge regarding these filarial worm’s life cycles such as pre-patent period, seasonality, host age and transmission routes. Corresponding metadata to our collected blood samples offers useful insights to elucidate these questions and, by using a DAS approach, ensure the retention of critical co-infection data. One pattern that has emerged in our study is that *S. yehi* is predominantly infecting calves and *R. andersoni* is infecting adults. This is also occurring in our DAS data between 2015 and 2018, where all samples, which were adults, tested positive for *R. andersoni* (Table S2). A possible explanation could be that *S. yehi* has higher mf, and thus higher transmission rates, in mammals with compromised or newly developing immunity (i.e., calves) (Nygren, 1990; Orro et al., 2006; Laaksonen et al., 2007, 2009b). The pre-patent period is considered to be 4 months for *S. tundra*, but unknown if this also applies to *S. yehi* (Laaksonen et al., 2009b). If so, these calves, which are about 8–9 months, should be old enough to have an established mosquito-transmitted infection. This precedent has been noticed in other *Setaria* species infections of cervids like black-tailed deer (*Odocoileus hemionus columbianus*), white-tailed deer and moose (Weinmann et al., 1973; Prestwood and Pursglove, 1977; Nygren, 1990; Laaksonen et al., 2007; Kutz et al., 2012). However, there is speculation that these juvenile hosts were infected *in utero* by L3s migrating from adult hosts to a susceptible fetus. This has been demonstrated in black-tailed deer with *S. yehi* infections and cattle hosts with other *Setaria* species from Asia (Shoho, 1965; Weinmann et al., 1973; Fujii et al., 1995; Wee et al., 1996; Kim et al., 2010).

On the other hand, *R. andersoni* is largely infecting adult moose. While not a characteristic of *Setaria*, some filarial species show higher filarial infection rates in adults rather than juveniles (Wisely et al., 2008), and this pattern has been demonstrated in previous work with *R. andersoni* (Grunenwald et al., 2016; Haake et al., 2024). This could be due to continual exposure to infection from vectors and thus increase the likelihood of transmission and higher worm burdens. In addition to largely infecting adults, there were 4 years (2015, 2016, 2017 and 2021) where moose were only positive for *R. andersoni*. While these data should be interpreted cautiously, it might be capturing a shift or cycling of the predominant filarial species in Kenai Peninsula. Indeed, environmental perturbations (e.g., climate change) can induce parasite host-shifting and/or disease outbreaks and one interpretation could be that the environmental changes are triggering a cascade effect to allow for the recent increase in *S. yehi* infections and co-infections. Such disturbances could bring unforeseen negative consequences to humans, animals and the environment. Overall, this study has expanded our understanding of the diversity, distribution and life cycle of neglected filarial nematodes infecting wild moose of Alaska. Adapting a DAS approach for mf screening has many advantages, especially in its use to detect co-infecting filarial species. Future work should focus on refining this molecular technique and application while also investigating filarial nematodes that were not, or could not, be detected through blood screening.

## Supporting information

10.1017/S0031182026101991.sm001 Kulpa et al. supplementary material 1 Kulpa et al. supplementary material

10.1017/S0031182026101991.sm002 Kulpa et al. supplementary material 2 Kulpa et al. supplementary material

10.1017/S0031182026101991.sm003 Kulpa et al. supplementary material 3 Kulpa et al. supplementary material

## Supplementary material

The supplementary material for this article can be found at https://doi.org/10.1017/S0031182026101991.

## Figure 1 Long description

The map of Kenai Peninsula, Alaska, illustrates three regions where moose blood was collected. The legend identifies these regions as GMU 15A, GMU 15B and GMU 15C. GMU 15A, the northernmost region, is marked with a dotted pattern. GMU 15B, the central region, is represented with a hatched pattern. GMU 15C, the southernmost region, is shown with a cross-hatched pattern. A scale bar at the bottom indicates distances in kilometers, ranging from 0 to 150. A compass rose on the left side indicates cardinal directions: north, east, south and west.

Navigate back to Figure 1.

## Figure 2 Long description

The image A showing a scatter plot titled Setaria yehi. The x-axis label is Proportion of mf density, with values from 0 percent to 100 percent. The y-axis label is Proportion of sequencing reads, with values from 0 percent to 100 percent. A dotted diagonal line runs upward from near the lower-left toward the upper-right. Text on the plot reads y equals 0.8556x plus 0.099 and R superscript 2 equals 0.6972. Multiple circular data points are plotted, including points near the upper-right close to 100 percent on both axes, several points in the upper range of the y-axis between about 80 percent and 100 percent with x-axis values between about 60 percent and 100 percent and several points along the bottom near 0 percent on the y-axis with x-axis values spread across the axis. The image B showing a scatter plot titled Rumenfilaria andersoni. The x-axis label is Proportion of mf density, with values from 0 percent to 100 percent. The y-axis label is Proportion of sequencing reads, with values from 0 percent to 100 percent. A dotted diagonal line runs upward from near the lower-left toward the upper-right. Text on the plot reads y equals 0.8556x plus 0.0455 and R superscript 2 equals 0.6972. Multiple circular data points are plotted, including several points near the left side with low x-axis values and y-axis values near 0 percent, several points near the top with y-axis values close to 100 percent and x-axis values between about 20 percent and 60 percent and several points near the right side with x-axis values close to 100 percent and y-axis values between about 20 percent and 60 percent.

Navigate back to Figure 2.

## Figure 3 Long description

A stacked bar graph titled MK vs DAS. X-axis label: none. X-axis categories: MK, DAS. Y-axis label: none. Y-axis range: 0 percent to 100 percent, with tick labels at 0 percent, 20 percent, 40 percent, 60 percent, 80 percent, 100 percent. Legend labels: SY plus, RA plus, CI plus, Negative. MK stacked bar: Negative 0 percent to 40 percent; CI plus 40 percent to 50 percent; RA plus 50 percent to 70 percent; SY plus 70 percent to 100 percent. DAS stacked bar: Negative 0 percent to 50 percent; CI plus 50 percent to 60 percent; RA plus 60 percent to 75 percent; SY plus 75 percent to 100 percent.

Navigate back to Figure 3.

## Figure 4 Long description

A stacked bar graph titled MK vs DAS left parenthesis by year right parenthesis. X axis categories: MK and DAS for 2019, MK and DAS for 2020, MK and DAS for 2021, MK and DAS for 2022. Y axis label: percent. Range: 0 percent to 100 percent. Legend labels: S Y plus percent, R A plus percent, C I plus percent, Negative. Plotted bars: 2019 MK: Negative about 35 percent; C I plus percent about 15 percent; R A plus percent about 10 percent; S Y plus percent about 40 percent. 2019 DAS: Negative about 40 percent; C I plus percent about 5 percent; R A plus percent about 20 percent; S Y plus percent about 35 percent. 2020 MK: Negative about 35 percent; C I plus percent about 10 percent; R A plus percent about 5 percent; S Y plus percent about 50 percent. 2020 DAS: Negative about 40 percent; C I plus percent about 5 percent; R A plus percent about 25 percent; S Y plus percent about 30 percent. 2021 MK: Negative about 30 percent; R A plus percent about 70 percent. 2021 DAS: Negative about 50 percent; R A plus percent about 50 percent. 2022 MK: Negative about 65 percent; C I plus percent about 10 percent; R A plus percent about 5 percent; S Y plus percent about 20 percent. 2022 DAS: Negative about 65 percent; C I plus percent about 10 percent; R A plus percent about 5 percent; S Y plus percent about 20 percent.

Navigate back to Figure 4.

## Figure 5 Long description

The stacked bar graph compares filarial species detection between MK and DAS by season, with vertical bars representing spring left parenthesis n equals 116 right parenthesis and fall left parenthesis n equals 22 right parenthesis. Each bar is divided into four categories: S Y plus, R A plus, C I plus and Negative, indicated by different patterns. In spring, MK shows a higher percentage of Negative samples compared to DAS, with S Y plus being the dominant positive category. In fall, both MK and DAS have a similar distribution, with Negative samples still being the largest category. The graph highlights that MK generally detects more Negative samples than DAS in both seasons, with slight variations in positive categories. The vertical axis is labeled percent, ranging from 0 to 100 percent.

Navigate back to Figure 5.

## Figure 6 Long description

A stacked bar graph titled MK v DAS left parenthesis by region right parenthesis. Y-axis label: percent. Range: 0 percent to 100 percent, with tick labels at 0 percent, 20 percent, 40 percent, 60 percent, 80 percent, 100 percent. X-axis categories: 15A left parenthesis n equals 55 right parenthesis, 15B left parenthesis n equals 67 right parenthesis, 15C left parenthesis n equals 16 right parenthesis. Each category has two stacked bars labeled MK and FIL. Legend categories: SY plus percent, RA plus percent, CI plus percent, Negative. 15A left parenthesis n equals 55 right parenthesis: MK bar segment values: Negative 35 percent; CI plus percent 10 percent; RA plus percent 25 percent; SY plus percent 30 percent. FIL bar segment values: Negative 40 percent; CI plus percent 5 percent; RA plus percent 25 percent; SY plus percent 30 percent. 15B left parenthesis n equals 67 right parenthesis: MK bar segment values: Negative 40 percent; CI plus percent 5 percent; RA plus percent 20 percent; SY plus percent 35 percent. FIL bar segment values: Negative 45 percent; CI plus percent 10 percent; RA plus percent 15 percent; SY plus percent 30 percent. 15C left parenthesis n equals 16 right parenthesis: MK bar segment values: Negative 70 percent; CI plus percent 0 percent; RA plus percent 20 percent; SY plus percent 10 percent. FIL bar segment values: Negative 75 percent; CI plus percent 0 percent; RA plus percent 15 percent; SY plus percent 10 percent.

Navigate back to Figure 6.

## Figure 7 Long description

A stacked bar graph titled MK vs DAS left parenthesis by age right parenthesis. Y axis label: percent. Y axis range: 0 percent to 100 percent, with tick labels at 0 percent, 20 percent, 40 percent, 60 percent, 80 percent, 100 percent. X axis categories, left to right: MK, DAS, MK, DAS. Group labels under the x axis: ADULT left parenthesis n equals 75 right parenthesis under the first two bars; CALF left parenthesis n equals 63 right parenthesis under the last two bars. Legend categories: SY plus percent, RA plus percent, CI minus I plus percent, Negative. Bar values are shown as stacked segments with boundaries but no numeric labels on the segments. ADULT group: 1. MK bar: Negative segment from 0 percent to about 45 percent; CI minus I plus percent segment from about 45 percent to about 55 percent; RA plus percent segment from about 55 percent to about 85 percent; SY plus percent segment from about 85 percent to 100 percent. 2. DAS bar: Negative segment from 0 percent to about 55 percent; CI minus I plus percent segment from about 55 percent to about 65 percent; RA plus percent segment from about 65 percent to about 85 percent; SY plus percent segment from about 85 percent to 100 percent. CALF group: 3. MK bar: Negative segment from 0 percent to about 40 percent; CI minus I plus percent segment from about 40 percent to about 55 percent; RA plus percent segment from about 55 percent to about 60 percent; SY plus percent segment from about 60 percent to 100 percent. 4. DAS bar: Negative segment from 0 percent to about 45 percent; CI minus I plus percent segment from about 45 percent to about 60 percent; RA plus percent segment from about 60 percent to about 65 percent; SY plus percent segment from about 65 percent to 100 percent.

Navigate back to Figure 7.

## Table 1 Long description

Counts of three S. yehi sequence variants are summarized for moose, grouped by year, season, region, and age. Overall totals are 34 for SV 1, 17 for SV 2, and 1 for SV 3. By year, detections peak in 2020 with 26 SV 1, 12 SV 2, and 1 SV 3; 2019 has 3 SV 1 and 2 SV 2, 2022 has 5 SV 1 and 3 SV 2, and 2021 has no detections. By season, spring accounts for nearly all observations with 31 SV 1, 15 SV 2, and 1 SV 3, while fall has 3 SV 1 and 2 SV 2. By region, 15A has 18 SV 1, 5 SV 2, and 1 SV 3; 15B has 15 SV 1 and 12 SV 2; 15C has 1 SV 1 only. By age, calves have 24 SV 1, 9 SV 2, and 1 SV 3, while adults have 10 SV 1 and 8 SV 2. The table reports counts within each grouping and does not indicate sampling effort or statistical significance across groups.

Navigate back to Table 1.

## Table 2 Long description

The table reports counts and percentages of moose blood samples classified as negative, positive for Setaria yehi, positive for Rumenfilaria andersoni, or co-infected, comparing Modified Knott’s test with deep amplicon sequencing across year, season, region, and age. Overall, deep amplicon sequencing shows fewer negatives than Modified Knott’s test and more co-infections. Totals: Modified Knott’s test reports 42.45 percent negative, 38.41 percent Setaria yehi, 27.54 percent Rumenfilaria andersoni, and 8.70 percent co-infections; deep amplicon sequencing reports 49.28 percent negative, 37.68 percent Setaria yehi, 26.09 percent Rumenfilaria andersoni, and 13.04 percent co-infections. By year, 2022 matches exactly between methods, while 2020 shows higher co-infections with sequencing than with Modified Knott’s test. By season, spring has higher co-infections with sequencing than with Modified Knott’s test, while fall co-infections are low for both methods. By region, co-infections are highest in 15A for both methods and lowest in 15C. By age, calves have higher Setaria yehi than adults, while adults have higher Rumenfilaria andersoni than calves; sequencing reports more co-infections than Modified Knott’s test in both age groups. Percentages within a row can sum to more than one hundred because co-infections overlap with single-species positives.

Navigate back to Table 2.
